# A New Approach to Examine Cell–Antibody Avidity with Surface Plasmon Resonance Imaging

**DOI:** 10.3390/bios15090559

**Published:** 2025-08-25

**Authors:** Richard B. M. Schasfoort, Elise van Doorn, Jos van Weperen, Anouk Mentink, Ruchi Bansal

**Affiliations:** 1 Department of Bioengineering Technologies, Technical Medical Centre, Faculty of Science and Technology, University of Twente, P.O. Box 217, 7500 AE Enschede, The Netherlandsr.bansal@utwente.nl (R.B.); 2 Vysens B.V., Fl. Hazemeijerstraat 800/A04, 7555 RJ Hengelo, The Netherlands; j.vanweperen@vysens.com

**Keywords:** surface plasmon resonance imaging, cell ligand interaction, avidity, ligand density gradient, tipping point, biosensor

## Abstract

In recent years, avidity has emerged as a critical parameter in antibody design, yet most current analytical instruments are limited to measuring affinity alone. This study aims to evaluate the capabilities and advantages of a novel surface plasmon resonance imaging instrument, CellVysion, designed to quantify cell–antibody avidity using a continuous antibody density gradient. A key feature of this approach is the identification of a “tipping point”—the specific ligand density, measured in µRIUs, at which cells remain bound to the sensor surface under defined shear flow conditions. In this paper, we present the technical principles and application of this method, demonstrating how avidity can be quantitatively assessed across different antibody–cell line combinations.

## 1. Introduction 

The effectiveness of antibodies as biotherapeutic drugs largely depends on their interactions with target cells [1], which is critical for treating several diseases, including cancer. Antibody function relies on complex interactions including associations with effector molecules and immune cells. While affinity refers to the strength of the interaction between a single antigen binding site and an antibody, avidity is the aggregate binding strength resulting from multiple interactions between an antibody and multiple antigenic epitopes [2]. Consequently, numerous low-affinity interactions can collectively yield high avidity [3]. There is substantial evidence demonstrating the crucial role of avidity in natural antibody biology, from antibody affinity maturation early in the immune response to the activation of effector functions following target engagement [4]. Recent studies increasingly emphasize the importance of avidity in shaping the interactions between antibodies and cells and antibody design [5]. 

A novel surface plasmon resonance imaging instrument called CellVysion has recently been developed to enable precise avidity measurements of cell–antibody interactions using a continuous gradient of ligand density [6]. This continuous antibody gradient is the unique core feature of the CellVysion, allowing for the accurate measurement of the binding strength (avidity) of cell–antibody interactions across a range of ligand densities [7]. The ligand density is quantitatively monitored at pixel locations within the channel. After generation of the continuous ligand density gradient, the injected cells sediment into the evanescent field and become visible in the SPR image. If the cell interacts with the immobilized ligands, it penetrates deeper in the evanescent field and can withstand a certain defined shear flow. The critical point at which cells remain bound under flow—the so-called tipping point—can be visualized in the SPR image. The ligand density at the tipping point, calculated in micro refractive index units (µRIUs) or picograms per square millimeter (pg/mm^2^), is characteristic of a specific antibody–cell line combination. 

The tipping point shifts with increasing shear force and reflects both receptor density and monovalent affinity per cell. To minimize non-specific binding, the SPR sensor surface is coated with a non-crosslinked hydrogel [8], demonstrating no interaction even with high-avidity-adherent cells. At zero antibody density, cells are not retained under shear flow, confirming the specificity of binding along the continuous gradient.

## 2. Materials and Methods

### 2.1. SPR Imager

The CellVysion SPR imager (Vysens B.V., Hengelo, The Netherlands) is an instrument in the Kretschmann–Raether configuration [9] and applies an automated angle set to operate the instrument at a fixed angle at the left flank of the SPR curve with pixel resolution. Any drift, skew, or vibration is removed. The reflectivity of all pixels is monitored instantly and a histogram of the number of pixels as a function of the pixel intensities is generated using the camera software. Then an optimal shift in the SPR angle is set automatically at the left flank of the SPR dip for the highest sensitive operation close to the inflection point [10] for all regions of interest (ROIs) simultaneously. The pixel intensities are calibrated to µRIUs using automated multiple injections of a refractive index calibrator and fitting the intensities for each pixel of the camera at any location in the SPR image. Now at any location, the shift in terms of pg/mm^2^ biomolecular accumulated mass can be measured. Standard data processing using the CRAZE principle (calibrating, referencing, aligning, zeroing, and exporting) is applied to show the biomolecular interaction at any location in the channel on the SPR sensor.

### 2.2. Cells

LNCaP (CRL-1740) and NCI-H1792 (CRL-5895) were purchased from the ATCC (Manassas, VA, USA) and LCL (GM12882) from the Coriell Institute (Camden, NJ, USA). The LNCaP and NCI-H1792 cell lines were cultured in RPMI1640 (Lonza, Basel, Switzerland) supplemented with 10% FBS (Sigma-Aldrich, St. Louis, MO, USA) and 1% penicillin/streptomycin (Lonza, Basel, Switzerland). For the LCL cells, the same medium was used but with 15% FBS instead. Furthermore, we investigated the influence on avidity of IgG glycosylation on Fcγ receptor (FcγRIIa) binding. When red blood cells (RBCs) were opsonized with anti-D antibodies (anti-Rhesus factor (Rh)), it means that the RBCs were coated with specific antibodies, which mark them for recognition by the immune system. The biotinylated FcγR gradient (FcγRIIa) was generated and after calibration, the ligand density along the channel was determined. RBCs were opsonized with three concentrations in dilutions of 10, 2.5, and 0.62 µg/mL (factor 4 diluted). The effect of opsonization of RBC’s binding with respect to the tipping point shift on the FcγRIIa gradient was determined.

### 2.3. Fluidics 

**Figure 1:**
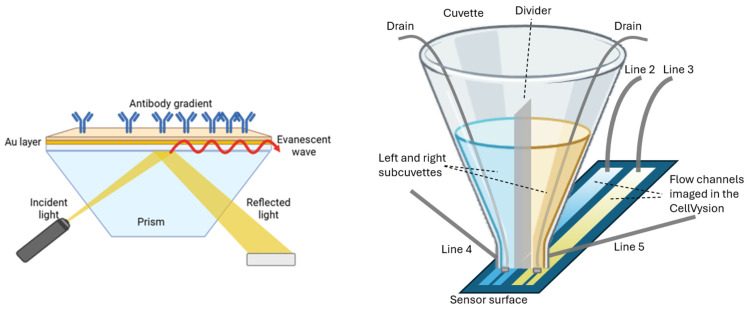
A schematic presentation of the SPR imager and the cuvette injection flow (CIF) fluidics, which is mounted on top of the sensor surface in the CellVysion. The reflectivity of the sensor surface can be imaged instantly in the SPR imager in reversed Kretschmann configuration [11]. Two parallel flow channels are connected to a cuvette with two separate sub-cuvettes (100 µL). The outlets (line 2 and 3) of the left and right flow channel are connected to a multichannel syringe pump of the CellVysion instrument. Lines 4 and 5 are applied to inject the ligand density gradient. The CIF with sub-cuvettes enables us to wash both channels simultaneously with larger volumes than 100 µL while operating the left and right channel independently via ligand or cell suspension injection in either the left or right sub-cuvette. The sub-cuvettes can be drained simultaneously. The CIF fluidics enable the generation of a continuous ligand density gradient by exposing the ligand to locations further away from the cuvette injection inlet in a timely manner.

The CellVysion SPR imager of Vysens B.V. Hengelo, The Netherlands, applies a valveless injection of samples in a so-called cuvette injection flow (CIF) cell (Figure 1). Furthermore, it enables the generation of two parallel ligand density gradients on the sensor surface. “Back-and-forth” flow-based fluidics enables unlimited interaction times using 70 μL of the sample. The CIF of the SPR imager enables the injection of cell suspensions directly from a cuvette placed on top of a two-channel flow cell. Without applying an air bubble to separate the running buffer from the sample, the cell suspension can be injected instantly into the flow chamber without delay. Large particles (e.g., cells or cell aggregates) can be aspirated without clogging the fluidics. The down position of the optics allows for subsequent sedimentation of the cells. To keep the sample volume low (e.g., 50 µL), back-and-forth flow was applied to reduce the sample volume. The CIF can be applied to generate a ligand density gradient in both flow channels.

In the SPR imager, the gradient enables the availability of all ligand densities along the channel, from very high to very low up to zero. The zero-ligand density location in a channel was used as a reference and serves as a negative control to compensate for common mode signals such as bulk refractive index shifts, temperature effects, and nonspecific binding to the hydrogel. In principle, it is possible to obtain the single molecular affinity parameters [12] from thousands of biomolecular interaction events on the continuous gradient.

### 2.4. Sensor Immobilization

**Figure 2:**
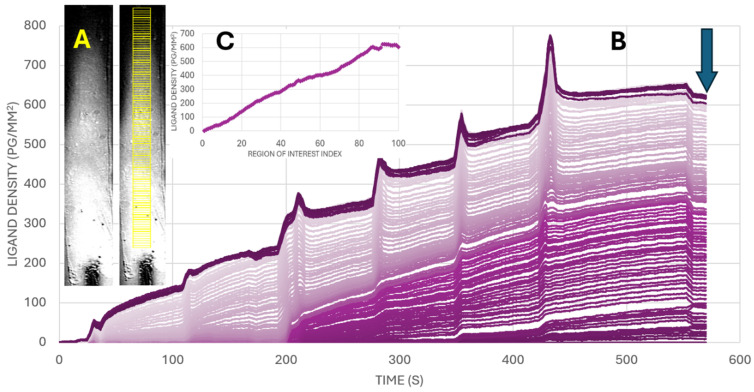
(**A**): The SPR images show the intensity profile without (left) and with (right) 100 ROIs after generating the continuous ligand density gradient. (**B**): Sensorgram obtained from 100 ROIs at concatenated locations in the flow cell. The continuous gradient generation on the sensor surface can be followed in real time. After calibrating the intensity (to µRIU values), the ligand density at the ROIs can be determined in pg/mm^2^. The final ligand density at the ROIs relevant for avidity determination can be determined after washing the sensor surface (see arrow at 560 s). (**C**): The resulting ligand density at the 100 ROIs located at the various positions in the flow channel show a close to linear response level from zero till about 600 pg/mm^2^ of immobilized antibody.

A high-quality streptavidin sensor (Xantec Bioanalytics GmbH, Düsseldorf DE) with a linear hydrogel (HC200M Strep) and reduced non-specific binding was used to immobilize biotinylated ligands through controlled and time-dependent exposure. A continuous gradient was created by injecting the ligand that flows through the microfluidic channel whereby ligands entering the channel earlier have more time to bind, while those reaching further downstream have progressively less time for interaction. Due to limited diffusion and flow dynamics, the ligand does not reach the end of the channel, leaving a distal region with zero ligands serving as a reference (negative control). Surface blocking is achieved using a standard biotin-free blocking buffer following ligand capture to minimize non-specific binding. Additionally, the SPR dip—the incident angle scan curve—is acquired at the beginning of the experiment, and the optimal SPR angle is set across all regions of interest (ROIs) using a histogram-based approach. ROIs (yellow borders as shown in the SPR image on the left of Figure 2A) were placed on the image stack recorded from the flow channels (i.e., 600 images recorded for an interval time of, e.g., 1 s for a 10-min sensorgram). The intensities of the pixels within an ROI were averaged, and the biomolecular interaction was followed during the continuous gradient generation process. By applying a convective flow in six consecutive steps, the ligand density gradient was generated and measured in real time (see Figure 2).

### 2.5. Tipping Point Measurement 

The gradient was generated on the sensor surface with the antibody of interest in a concentration of 20 µg/mL. A higher ligand (antibody) concentration generates a steep gradient, while a lower concentration will not generate a saturation value at the injection site on the sensor surface; hence, a ligand (antibody) concentration was optimized to generate a continuous gradient. For measuring high-avidity cell interactions, a low level of ligand density is required and therefore a non-saturated surface level is preferable to generate the lowest steepness for accurate high-avidity cell interactions. As always, a zero-ligand density should be present on the sensor surface for referencing purposes. After the generation of the antibody gradient, 50 µL of the cell suspension was injected with a concentration of 5 × 10^6^ cells/mL. A lower cell concentration was also used; however, the disadvantage was that less cells landed on the sensor surface at the important region close to the tipping point, compromising the accurate avidity measurements. The cells were kept in running buffer consisting of PBS, 1% BSA, 0.25% EDTA, and 0.01% Tween-20. These cells were sedimented for 10 min, followed by a wash of the sensor surface with increasing flow speeds in seven steps from 1, 2, 5, 10, 20, and 40 up to 80 µL/s. After the washing steps, the tipping point could be determined for a certain flow rate, which gives the characteristic ligand density for the cell–antibody pair. For low-avidity blood cells, the tipping point was determined at 5 µL/s, while this was 80 µL/s for high-avidity-adherent cells. 

## 3. Results

In the SPR imager, the generation of the gradient can be followed in real time. Antibodies were successfully and reliably immobilized on the sensor surface using a continuous gradient, as can be visualized at various locations in the flow channel (see Figure 2A). At the end of the process of generating a gradient (see arrow in Figure 2B), the ligand density can be determined. In Figure 2C, the ligand density as a function of the ROI location in the flow channel is shown.

After generating the continuous ligand density gradient, a suspension of cells was injected via the CIF cell into the SPR imager. The cells were allowed to sediment onto the sensor surface before being subjected to increasing flow speeds. The tipping point—the characteristic antibody density required to retain the cells under a defined shear flow—served as a quantitative measure of the cell avidity. Owing to its label-free and real-time detection capabilities for measuring avidity effects, SPR imaging enables a rapid and user-friendly assay that functions in a nearly plug-and-play fashion, offering significant advantages in ease of use and throughput compared to alternative methods. Other techniques include ELISA, LigandTracer [13], and the z-Movi Cell Avidity Analyzer. The latter uses acoustics to measure forces and interactions between cells, providing insights into cell–cell interaction and avidity [14,15]. The CellVysion SPR imager provides real-time, label-free, and kinetic measurement of antibody–cell interaction, making it a powerful tool for characterizing avidity and optimizing antibody-based therapies.

Importantly, the avidity measurement with the CellVysion SPR imager is time-efficient. The entire process, from the generation of a continuous gradient (10 min) to cell sedimentation (10 min) to shear flow application in a stepwise manner (8 min), can be completed in under 30 min.

Four different cell types were tested; blood cells were opsonized with three different concentrations of anti-D antibody (αD) binding to the FcγRIIa gradient. These included the non-adherent cell line LCL on two different antibody gradients and the adherent cell lines, NCI-H1792 and LNCaP, both on an anti-EpCAM antibody gradient. The ligand density at the tipping points was determined in seven cell–ligand combinations. Low avidity binding RBCs opsonized with 0.62 μg/mL αD showed a tipping point at 610 pg/mm^2^ FcγRIIa receptor and higher opsonizations showed higher avidity: 2.5 μg/mL αD opsonized RBC had a tipping point at 340 pg/mm^2^ and 10 μg/mL αD opsonized RBC was at 300 pg/mm^2^. The other cell line antibody gradient combinations show higher avidity and, therefore, less antibody density is needed for capturing the cells at the tipping point. The LCL–αCD19 gradient showed a tipping point at 81 pg/mm^2^, while the same cell line LCL–αCD45 showed a tipping point at 27 pg/mm^2^. The other two cell lines, NCI-H1792 and LNCaP, possess a different number of EpCAM receptors. LNCaP showed the highest number of EpCAM receptors. Using NCI-H1792 cells, the anti-EpCAM (VU1D9) gradient generated a tipping point at 44 pg/mm^2^, while LNCaP showed higher avidity and the tipping point is already reached at 7 pg/mm^2^.

LNCaPs showed the highest avidity towards the anti-EpCAM gradient than any other tested cell line tested with the CellVysion SPR imager. The optimized HC200M sensor (Xantec Bioanalytics, Düsseldorf, Germany) surface prevented the non-specific binding of cells, as no binding was observed at zero-ligand density for all tested cells. Altogether, we showed that the CellVysion can be used to examine the avidity using adherent and non-adherent cell lines.

## 4. Discussion

A new surface plasmon resonance (SPR) imager is introduced here for the first time, featuring a unique continuous ligand density gradient on the sensor surface. The system enables label-free and time-resolved monitoring of antibody–cell interactions at the single-cell level, offering direct quantification of avidity rather than only affinity.

**Figure 3:**
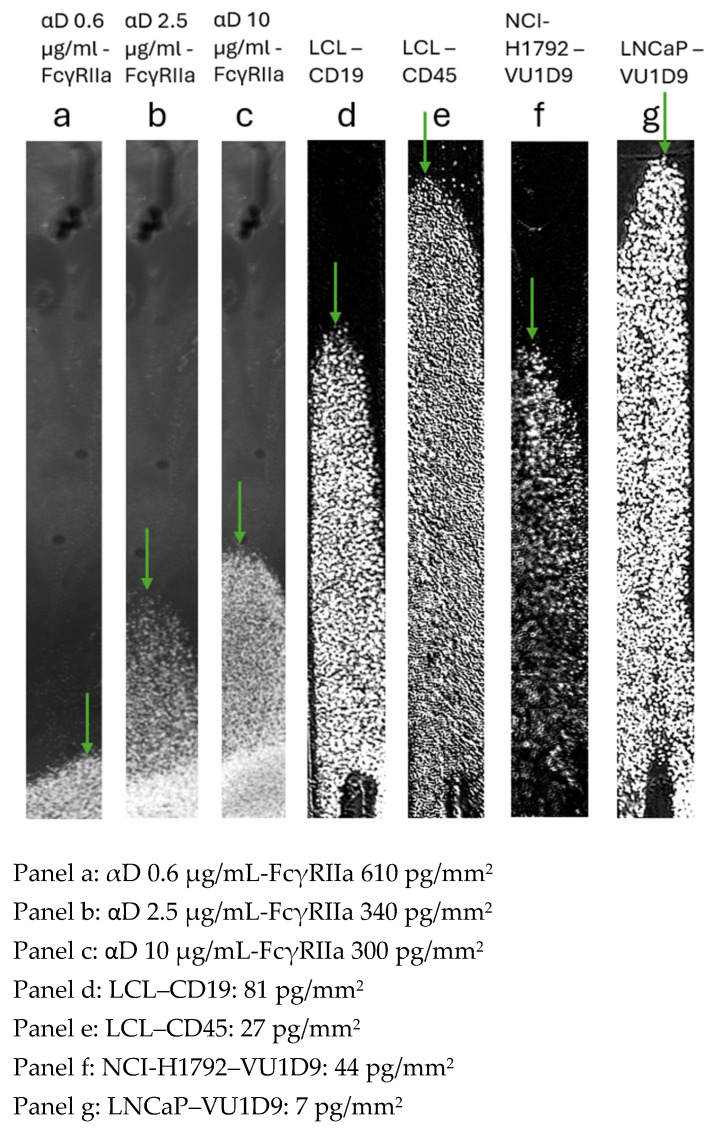
Four different cell types were tested. Panel a, b, c: Blood cells opsonized with different concentrations of anti-D antibody (anti-Rhesus factor); panel d and e: The non-adherent cell line LCL with CD19 and with CD45; panel f and g: The adherent cell lines, NCI-H1792 and LNCaP, with an anti-EpCAM VU1D9 antibody gradient. A tipping point (indicated with green arrows) at low ligand density measured at high shear force indicates high cell–antibody avidity. LNCaP cells showed highest avidity towards an anti-EpCAM gradient. The blood cells show low avidity and were tested with slow shear flow of 10 μL/s, while the other cell lines were exposed to shear flow of 80 µL/s. The instrument automatically applies increasing flow rates of 1, 2, 5, 10, 20, 40, and 80 µL/s.

The ligand density gradient is generated in six concatenated flow steps across the sensor surface (Figure 2). Biotinylated antibodies are introduced at the inlet of the lateral flow channel where they bind to the streptavidin-coated sensor. Antibody diffusion from the bulk solution to the sensor surface in the flow channel will occur. This results in a decreasing concentration of antibody in the bulk solution due to which progressively fewer antibodies will diffuse to the sensor surface deeper in the channel. During the change in the flow conditions, the antibody solution is injected deeper in the flow channel, and we observed peaks in refractive index caused by hydrodynamic changes at these transitions. The effect is more pronounced at higher ligand densities likely due to the hydrogel and immobilized antibodies being pressed deeper into the evanescent field during flow perturbation. The regions of interest (ROIs) located close to the inlet show the highest binding, while the ROIs at the end of the channel show negligible to no binding, confirming a well-defined gradient. As can be observed in Figure 2, the immobilized antibody density varies almost linearly from ROI #1 to ROI #83, reaching up to approximately 600 pg/mm^2^. To get sufficient resolution in avidity, it is important to tune the steepness of the antibody gradient by injecting the right biotinylated antibody concentration for building the antibody density gradient. In other words, it is not always needed to use the full capacity of the streptavidin-coated sensor surface. If the steepness is too high, then a lower concentration of ligand during the generation of the gradient should be injected to achieve reliable spatial resolution. As can be seen in Figure 3, the range with a gradual increase in ligand density is from ROI #1 till ROI #83. The higher numbered ROIs show a saturation of the ligand density. We show here optimally tuned fluidics to obtain the lowest steepness of increasing ligand density. Tuning the injected concentration will result in changed responses. Also, the timing of exposure and fluidic conditions will lead to changes in the resulting ligand density gradient. For measuring the avidity of cell binding in terms of ligand density at the tipping point, the final ligand density in pg/mm^2^ (see arrow in Figure 2) is important after washing the sensor surface with buffer. The process of building the continuous gradient in time can be neglected, and only the final level of antibodies in pg/mm^2^ values counts for binding of the cells.

When there is no specific cell receptor or specific ligand present, the cells will be visible only during the sedimentation phase but are washed immediately from the sensor after starting the flow [16]. Because we apply a continuous gradient up to zero, there is no need to use the other channel as a reference channel. In the channel, there is always a part where no ligands are immobilized, and this part can be used as a reference for common mode effects as bulk refractive index changes, temperature changes, hydrogel shrinkage, etcetera. As observed previously [17], cells that bind to immobilized antibodies penetrate deeper into the hydrogel under shear flow, increasing their SPR signal. This behavior correlates with mechanical stabilization of the binding under flow. Therefore, increasing the shear force shifts the tipping point to higher ligand densities, indicating that higher avidity is needed to resist detachment. When the shear force is increased by changing the flow rate, the tipping point moves to higher ligand density. So, more avidity is needed to withstand the higher shear force. However, this effect of shift in tipping point depends on the number of receptors and the monovalent affinity of receptor–ligand binding.

Recent studies have demonstrated that specific modifications of the glycosylation of the Fc gamma receptor (FcγR) of erythrocytes, especially the removal of fucose (afucosylation), dramatically enhance FcγRIII binding, thereby potentiating natural killer (NK) cell responses [18,19]. Complementary to these molecular studies, innovative SPR imaging systems enable the real-time, label-free measurement of such interactions [20]. In this study, we investigated the binding of red blood cells (RBCs) opsonized with anti-D (anti-Rh) antibodies to immobilized Fcγ receptor (FcγRIIa) gradients under defined shear conditions using the CellVysion system. The anti-D antibodies are typically IgG antibodies that target the D-antigen on the surface of RBCs [21]. When RBCs are opsonized with anti-D antibodies (anti-Rhesus factor, Rh), it means the RBCs are coated with antibodies, which mark them for recognition by the immune system. The relevance of understanding the role of FcγRs in the binding of opsonized RBCs is critical in clinical settings, particularly in the management of Rh disease and autoimmune hemolytic anemia [22]. Red blood cells (RBCs), when opsonized with anti-D IgG, serve as a clinically relevant model system, particularly in contexts such as Rh disease.

A feasibility study at Sanquin Research (Amsterdam, NL) provides deeper insights into the mechanisms of glycosylation for the binding of anti-D opsonized RBCs to the FcγRIIa [23] continuous gradient. We observed differences in avidity when the degree of opsonization of RBCs with anti-D changed, as can be observed in Figure 3 panel a, b, and c. So, RBCs show low avidity and therefore need higher ligand density to bind to the sensor surface. We apply a shear force induced by 10 µL/s because with higher shear forces, all cells were washed from the sensor surface and a tipping point was not created. In contrast, higher opsonization concentration of anti-D led to stronger binding at lower ligand densities (Figure 3, panels a–c).

In contrast to RBCs, non-adherent lymphoblastoid cells (LCLs) exhibited higher avidity (Figure 3, panels d–e). While receptor expression levels (CD19 and CD45) [24,25] did not differ substantially, the increased avidity likely reflects differences in single molecular binding affinity per receptor–ligand interaction. Thus, both parameters contribute to overall avidity as follows: (1) receptor density (number of receptors on the cell surface) and (2) monovalent binding affinity of individual receptor–ligand interactions. The characteristic ligand density at the tipping point reflects the measure of both parameters and serves as a reliable avidity metric. Finally, tumor cell lines NCI-H1792 and LNCaP demonstrated exceptionally strong adhesion (Figure 3, panels f–g), remaining bound even at very low ligand densities under high shear force. Preliminary flow cytometry data confirms that the number of EpCAM receptors of LNCaP cells [26] are at least 10 times more than for the NCI-H1792 cells. Since the expression of certain markers on cell lines can vary between different labs, we have tested this on the cell lines cultured in our lab [27,28]. This explains why we observe a shifted tipping point for LNCaP versus NCI-H1792 to the same continuous anti-EpCAM gradient.

These findings suggest that CellVysion can resolve differences in avidity between diverse cell types and antibody formats.

## 5. Conclusions

In this paper, we present a new SPR imager, CellVysion, with the capability of measuring antibody–cell avidity for the first time. The SPR imager features a unique continuous antibody density gradient across the sensor surface generated for highly sensitive and spatially resolved avidity measurements. A new functional parameter of avidity is introduced, which is the characteristic ligand density value just at the point where the cells can still withstand a fixed shear flow condition. This so-called tipping point can be measured accurately and depends on the receptor density and the monovalent affinity between the antibody and receptor. A tipping point at low ligand density and high shear force corresponds to high overall cell–antibody avidity.

As demonstrated, measuring cell–antibody avidity in terms of the antibody density at the tipping point is extremely sensitive compared to other techniques. Here, we successfully evaluated a broad range of avidity values from several cell lines based on the number of cell receptors per cell, such as opsonized blood cells and LCL cells with two different receptors, NCI-H1792 and LNCaP, by using various antibodies and receptor combinations.

Importantly, CellVysion allows for fine-tuned control of the ligand density gradient (ranging in this paper from ~7 pg/mm^2^ to ~610 pg/mm^2^) and shear force (from 1 to 80 µL/s), covering a wide dynamic range of avidity interactions. This combination of real-time, label-free detection with spatial and mechanical resolution represents a major advancement over existing technologies for studying cell–antibody interactions.

In summary, CellVysion introduces a powerful new method for measuring cellular avidity, with potential applications in therapeutic antibody development, including ADC [29], ADCC [30], bi-specific [31] and mini-antibodies [5], immunophenotyping, CAR-T therapy [32], and biomarker discovery [33].

## Data Availability

Data is contained within the article.
